# Catheter-Based Therapies Decrease Mortality in Patients With Intermediate and High-Risk Pulmonary Embolism: Evidence From Meta-Analysis of 65,589 Patients

**DOI:** 10.3389/fcvm.2022.861307

**Published:** 2022-06-16

**Authors:** Arkadiusz Pietrasik, Aleksandra Gąsecka, Łukasz Szarpak, Michał Pruc, Tomasz Kopiec, Szymon Darocha, Marta Banaszkiewicz, Maciej Niewada, Marcin Grabowski, Marcin Kurzyna

**Affiliations:** ^1^1st Chair and Department of Cardiology, Medical University of Warsaw, Warsaw, Poland; ^2^Research Unit, Maria Sklodowska-Curie Białystok Oncology Center, Białystok, Poland; ^3^Institute of Outcomes Research, Maria Sklodowska-Curie Medical Academy in Warsaw, Warsaw, Poland; ^4^Research Unit, Polish Society of Disaster Medicine, Warsaw, Poland; ^5^Department of Pulmonary Circulation, Thromboembolic Diseases and Cardiology, Centre of Postgraduate Medical Education, European Health Centre Otwock, Otwock, Poland; ^6^Department of Experimental and Clinical Pharmacology, Medical University of Warsaw, Warsaw, Poland

**Keywords:** pulmonary embolism, catheter-based therapies, meta-analysis, pulmonary embolism response team, PERT

## Abstract

**Background:**

Catheter-directed therapies (CDT) are an alternative to systemic thrombolysis (ST) in pulmonary embolism (PE) patients, but the mortality benefit of CDT is unclear.

**Objective:**

We conducted a systematic review with meta-analysis to compare the efficacy and safety of CDT and ST in intermediate-high and high-risk PE.

**Methods:**

We included (P) participants, adult PE patients; (I) intervention, CDT; (C) comparison, ST; (O) outcomes, mortality, complications, in-hospital treatment, and length of hospital stay; (S) study design, randomized controlled trials (RCTs), or cohort comparing CDT and ST. The primary endpoint was 30-day mortality. Secondary outcomes included treatment-related complications including bleeding, the use of hospital resources, and length of hospital stay.

**Results:**

Eleven studies including 65,589 patients met the inclusion criteria. Thirty-day mortality was lower in the CDT group, compared to ST group [7.3 vs. 13.6%; odds ratio (OR) = 0.51, 95% confidence interval (CI) 0.38–0.69, *p* < 0.001]. The rates of myocardial injury, cardiac arrest, and stroke were lower in CDT group, compared to ST group (*p* < 0.001 for all). The rates of any major bleeding, intracranial hemorrhage, hemoptysis, and red blood cell transfusion were lower in patients treated with CDT, compared to ST (*p* ≤ 0.01 for all). Extracorporeal life support was used more often in patients treated with CDT, compared to ST (0.5 vs. 0.2%, OR = 2.52, 95% CI 1.88–3.39, *p* < 0.001). The use of hospital resources and length of hospital stay were comparable in both groups.

**Conclusion:**

CDT might decrease mortality in patients with intermediate-high and high-risk PE and were associated with fewer complications, including major bleeding.

## Introduction

Acute pulmonary embolism (PE) is the third most frequent cause of cardiovascular morbidity and mortality worldwide (1). Whereas anticoagulation is the mainstay of therapy for low-risk and intermediate-low risk PE patients, the choice of the therapy for patients with intermediate-high and high-risk PE is more complex (2). Systemic thrombolysis (ST) is approved for high-risk PE, but its use for intermediate-risk patients remains uncertain (2, 3). Although ST improves pulmonary perfusion and right ventricle function in patients with PE, it is associated with an overall 10% risk of major bleeding and a 3–5% risk of intracranial hemorrhage (4). In addition, there are numerous patients in whom ST is contraindicated or has failed. Catheter-directed therapies (CDT) are an alternative treatment option for intermediate-high and high-risk PE and for patients with contraindications to ST. CDT are all therapies which are delivered *via* a catheter placed in the branches of the pulmonary artery directly into the thrombus. They include intrapulmonary administration of low doses of thrombolytic drugs, ultrasound-assisted thrombolysis, mechanical aspiration thrombectomy, and direct clot retrieval systems (5–7). CDT efficiently alleviate right ventricle overload and allow to decrease the dose of thrombolytic drug administered (e.g., local or ultrasound-assisted thrombolysis), or even to entirely avoid thrombolytic drug administration (e.g., thrombectomy-based methods). Preliminary results of registry-based studies and case series suggest the CDT procedural success rate of above 80%, which is defined as improvement in right ventricle function, hemodynamic stabilization, correction of hypoxemia, and survival to hospital discharge. In addition, the rate of major bleeding complications might be reduced in CDT, compared with ST. However, the clear mortality benefit of CDT remains to be demonstrated (8–11). Therefore, we conducted a systematic review with meta-analysis to compare the efficacy and safety of CDT and ST in patients with high and moderate-to-high PE.

## Methods

The current systematic review and meta-analysis were conducted according to Preferred Reporting Items for Systematic reviews and Meta-analysis (PRISMA) statement.

### Search Strategy

A systematic review was carried out using PubMed, Web of Science, SCOPUS, EMBASE, and Cochrane Collaboration Databases electronic databases for relevant articles published through June 5, 2021, using the following keywords: “pulmonary embolism” OR “PE” OR “embolus” AND “catheter-based therapies” OR “thrombolysis” AND “systemic thrombolysis.” All retrieved articles were reviewed by two authors (ŁS and AG) independently. Any disagreement was resolved through consensus or, if necessary, by discussion with a third author (AP). We restricted articles to published in English language. The reference lists of eligible studies were also manually searched to identify any additional relevant studies. All references were saved in an EndNote (End Note, Inc., Philadelphia, PA, United States) library used to identify the duplicates.

### Inclusion and Exclusion Criteria

The PICOS strategy consisting of patient, intervention, comparison, and the outcome was used as a tool to ensure focused clinical questions. The prespecified criteria for studies included in the meta-analysis were as follows: (P) participants, adult patients with PE; (I) intervention, CDT; (C) comparison, ST; (O) outcomes, detailed information for mortality, complications, in-hospital treatment, and length of hospital stay; (S) study design, randomized controlled trials (RCTs), or cohort comparing CDT and ST for their effects on outcomes in patients with PE.

### Data Extraction

The titles and abstracts were screened for relevance by two authors (MP and TK) independently; if differences were found, they were discussed until a consensus was reached. The manuscripts of selected titles and abstracts were assessed for inclusion, and the authors were contacted if further information was required. Using the selection criteria enlisted above, the three reviewers (AP, AG, and ŁS) independently identified the papers to be included and excluded, and data from the included papers were extracted using pre-defined extraction flow sheets. The following information was extracted: authors, year of publication, study design, number of patients enrolled, patient characteristics, mortality outcomes, complications, and length of hospital stay.

### Quality Assessment

Two investigators (ŁS and AG) independently extracted individual study data and evaluated studies for risk of bias. Any disagreements were discussed and resolved in a consensus meeting with the third reviewer (AP). Data heterogeneity and methodological quality of the included studies were assessed using The Newcastle–Ottawa Scale (12). The scale is divided in three different sections: study selection; comparability and verification of exposure; and outcome investigated. We analyzed questions from each section to receive a star/point. According to the number of points received, cohort studies were categorized as follows: (1) high risk of bias—up to 3 points; (2) moderate risk of bias—4–6 points and; (3) low risk of bias—7–9 points; and cross-sectional studies were categorized as following: (1) high risk of bias—up to 4 points; (2) moderate risk of bias—5–6 points; (3) low risk of bias—7–8 points; and (4) very low risk of bias—9–10 points.

### Outcomes

The primary endpoint was 30-day mortality after the index PE episode. Secondary outcomes included treatment-related complications including bleeding, the use of hospital resources [admission to intensive care unit (ICU), invasive mechanical ventilation, use of extracorporeal life support (ECLS), inferior vena cava (IVC) filter insertion], and length of hospital stay.

### Statistical Analysis

Statistical analysis was performed with Review Manager (RevMan) software, version 5.4 (Cochrane Collaboration, Oxford, United Kingdom). The Mantel–Haenszel method was used to analyze dichotomous outcomes, and the results were reported as odds ratio (ORs). For continuous measures, we calculated the standardized mean differences (MD). A random-effect model was applied to analyze the data. The results of the meta-analysis are presented as ORs with 95% confidence intervals (CI) for dichotomous measures. When the continuous outcome was reported in a study as median, range, and interquartile range, we estimated means and standard deviations using the formula described by Hozo et al. (13). We quantified heterogeneity in each analysis by the tau-squared and *I*^2^ statistics. Heterogeneity was detected with the chi-squared test with *n*-1 degree of freedom, which was expressed as *I*^2^. Values of *I*^2^ > 50 and >75% were considered to indicate moderate and significant heterogeneity among studies, respectively. A two-tailed *p*-value less than 0.05 was considered statistically significant.

## Results

### Study Selection and Characteristics

**FIGURE 1:**
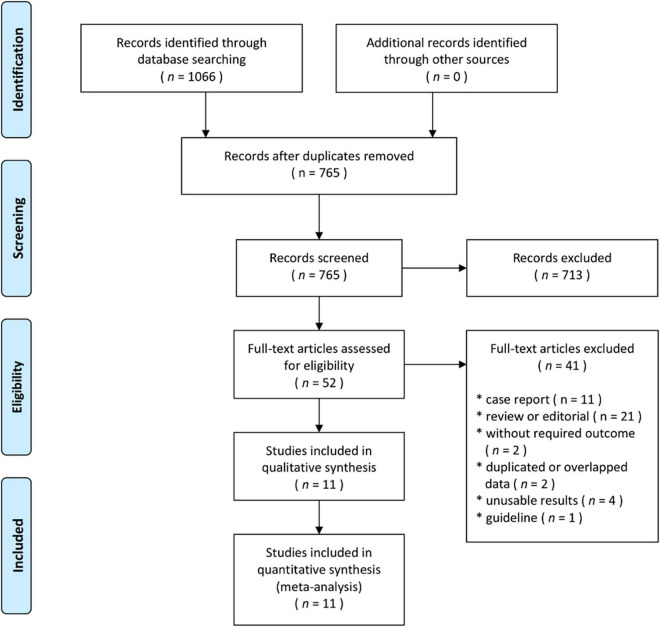
Flow diagram showing the stages of database searching and study selection according to the PRISMA guidelines.

The flow diagram showing the stages of database searching and study selection according to the PRISMA guidelines is shown in Figure 1. Of the 765 unduplicated records that were identified during our initial search, the full texts of 52 articles were fully reviewed. Eleven articles met the inclusion criteria and were included in the systematic review. A total of 10 non-randomized controlled trials (RCTs) (13–22) and one RCT (7) published between 2014 and 2021 including 65,589 participants were analyzed. In the included studies, 25,654 patients were treated with CDT and 39,935 were treated with ST. Nine studies were conducted in the United States (13–21), one study in Germany (7), and one study in the South Korea (22). The study characteristics and patient baseline characteristics are shown in Table 1. Detailed inclusion and exclusion criteria, primary outcomes, and findings of individual studies are shown in Supplementary Table 1. According to the Newcastle–Ottawa Scale, the risk of bias was considered very low (7) or low (14–22) (Table 1).

**TABLE 1:** Study characteristics and patient baseline characteristics.

Study	Country	Study design	CDT type	PERT	CDT			ST			NOS score
						
					No of patients	Age	Sex, male	No of patients	Age	Sex, male	
Beyer et al. (18)	United States	Retrospective study	Catheter-directed thrombolysis, US-assisted thrombolysis	NS	2,060	60.0 ± 15.2	1,045 (50.7%)	3,376	59.2 ± 15.9	1,724 (51.1%)	8
D’Auria et al. (17)	United States	Retrospective study	Catheter-directed thrombolysis, US-assisted thrombolysis	NS	99	57.8 ± 12.2	46 (46.5%)	240	62.5 ± 13.7	121 (50.4%)	8
Geller et al. (20)	United States	Retrospective study	Catheter-directed thrombolysis	NS	632	57.2 ± 16.2	344 (54.4%)	1,283	57.4 ± 16.3	652 (50.9%)	7
Harrison et al. (15)	United States	Retrospective study	Catheter-directed thrombolysis	Yes	18	73.8 ± 6.19	NS	108	75.5 ± 7.99	NS	8
Hennemeyer et al. (13)	United States	Retrospective study	US-assisted thrombolysis, aspiration thrombectomy	NS	36	59 ± 15.93	17 (47.2%)	43	60 ± 17.16	20 (46.5%)	7
Khaing et al. (20)	United States	Retrospective study	Catheter-directed thrombolysis, aspiration thrombectomy	Yes	16	50.5 ± 10.2	6 (37.5%)	30	61 ± 8.9	13 (43.3%)	8
Kucher et al. (7)	Germany	Multicenter randomized, controlled trial	US-assisted thrombolysis	NS	30	64 ± 15	11 (36.7%)	29	62 ± 13	17 (58.6%)	9
Patel et al. (16)	United States	Retrospective study	Catheter-directed thrombolysis	NS	352	59.59 ± 15.24	169 (48.0%)	1,169	58.44 ± 16.07	496 (42.4%)	8
Percy et al. (14)	United States	Retrospective study	Not specified	NS	22,336	62 ± 24	11,282 (50.5%)	33,553	58 ± 23	16,907 (50.4%)	8
Sharifi et al. (21)	United States	Retrospective study	US-assisted thrombolysis	NS	47	59 ± 14	27 (57.4%)	50	61 ± 13	29 (58.0%)	8
Yoo et al. (22)	South Korea	Retrospective study	Catheter-directed thrombolysis	NS	28	61.5 ± 17.3	13 (46.4%)	44	65.5 ± 16.8	9 (20.5%)	8

*CDT, catheter-directed therapies; NOS, Newcastle–Ottawa score; NS, not specified; PERT, pulmonary embolism response team; ST, systemic thrombolysis; US, ultrasound.*

Comparison of patient age, gender, and presentation with massive PE between the CDT and ST groups is shown in Supplementary Figures 1–3. There were no differences regarding the age of patients treated with CDT and ST (MD = -0.84, 95% CI -2.88–1.21, *p* = 0.42; Supplementary Figure 1) and the gender distribution (OR = 1.01, 95% CI 0.98–1.04, *p* = 0.52; Supplementary Figure 2). The percentage of patients who presented with massive PE was comparable in both groups (OR = 0.72, 95% CI 0.39–1.31, *p* = 0.28; Supplementary Figure 3). Comparison of the past medical history of patients treated with CDT and ST is shown in Supplementary Table 2. Diabetes mellitus (DM) was slightly less prevalent, whereas the previous stroke was more prevalent in the CDT group, compared to ST group (21.9 vs. 23.0%, *p* = 0.02 for DM; 19.2 vs. 15.0%, *p* = 0.04 for stroke, respectively). In CDT group, less patients presented with shock (8.0 vs. 17.2%, *p* < 0.001) and cardiac arrest (4.3 vs. 13.3%, *p* < 0.001), compared to ST group. The rates of other comorbidities were comparable between the groups.

### Quality Assessment

A summary of the risk of bias in the included studies is shown in Supplementary Figures 4–7. The one RCT had low overall risk of bias (7). Among non-randomized studies, five had overall low risk of bias (14, 16, 18–20), three moderate risk (13, 21, 22), and two high risk (15, 17). High risk of bias was mostly due to the selection of participants and missing data. Moderate risk of bias was due to the selection of participants, classification of the intervention, and measurement of the reported outcomes. Altogether, the majority of studies included in the meta-analysis had low risk of bias and thus high quality of data.

### Primary Outcome

Primary and secondary outcomes are shown in Table 2. In-hospital mortality was reported in seven studies and was lower in the CDT group compared to ST group (6.4 vs. 15.9%, respectively; OR = 0.40, 95% CI 0.30–0.55, *I*^2^ = 79%, *p* < 0.001). Thirty-day mortality (at 28 or 30 days) was reported in four studies and was lower in the CDT group, compared to ST group (7.3 vs. 13.6%; OR = 0.51, 95% CI 0.38–0.69, *I*^2^ = 18%, *p* < 0.001).

**TABLE 2:** Primary and secondary outcomes in patients treated with catheter-directed therapies (CDT) and systemic thrombolysis (ST).

Outcome	No of studies	Events/participants		Events		Heterogeneity between trials		*P*-value for differences across groups
				
		CDT group	ST group	OR	95% CI	*P*-value	*I*^2^ statistic	
**Mortality**								
In-hospital mortality	7	1,635/25,442 (6.4%)	6,279/39,563 (15.9%)	0.40	0.30–0.55	<0.001	79%	**<0.001**
28-d/30-d mortality	4	59/806 (7.3%)	220/1,617 (13.6%)	0.51	0.38–0.69	0.30	18%	**<0.001**
90-d mortality	2	4/66 (6.1%)	7/72 (9.7%)	0.66	0.19–2.28	0.61	0%	0.51
RCT	1	4/36 (11.1%)	6/43 (14.0%)	0.77	0.20–2.98	NA	NA	0.71
Non-RCT	1	0/30 (0.0%)	1/29 (3.4%)	0.31	0.01–7.96	NA	NA	0.48
1-yr mortality	2	82/731 (11.9%)	293/1,523 (19.4%)	0.46	0.24–0.87	0.11	61%	**0.02**
**Hospital resources**								
ICU admission	2	29/44 (65.9%)	43/74 (58.1%)	2.60	0.15–44.78	0.06	72%	0.51
Invasive mechanical ventilation	2	129/2,088 (6.2%)	652/3,420 (19.1%)	0.59	0.10–3.31	0.002	90%	0.55
ECLS application	2	117/22,364 (0.5%)	70/33,597 (0.2%)	2.52	1.88–3.39	0.54	0%	**<0.001**
IVC filter insertion	3	5/62 (8.1%)	7/182 (3.9%)	1.94	0.61–6.14	0.20	39%	0.26
**Complications**								
Acute myocardial infarction	1	963/22,336 (4.3%)	2,010/33,553 (5.9%)	0.71	0.65–0.77	NA	NA	**<0.001**
Acute kidney injury	2	3,157/22,688 (13.9%)	7,700/34,722 (22.2%)	1.36	0.19–9.82	0.003	89%	0.76
Complete heart block	1	65/22,336 (0.3%)	78/33,553 (0.2%)	1.25	0.90–1.74	NA	NA	0.18
Cardiac arrest	1	868/22,336 (3.9%)	3,764/33,553 (11.2%)	0.32	0.30–0.35	NA	NA	**<0.001**
Stroke	1	583/22,336 (2.6%)	1,982/33,553 (5.9%)	0.43	0.39–0.47	NA	NA	**<0.001**
30-day readmission	1	144/2,060 (6.9%)	323/3,376 (9.6%)	0.71	0.58–0.87	NA	NA	**0.001**
**Bleeding**								
Any bleeding complication	5	112/822 (13.6%)	156/1,647 (9.5%)	1.51	1.16–1.96	0.63	0%	**0.002**
Any major bleeding	3	2,911/24,424 (11.9%)	6,447/36,973 (17.4%)	0.61	0.53–0.70	0.17	44%	**<0.001**
Intracranial hemorrhage	5	182/25,479 (0.7%)	801/39,621 (0.1%)	0.56	0.24–1.30	<0.001	83%	0.18
Gastrointestinal bleed	3	188/2,791 (6.7%)	445/4,899 (9.1%)	0.96	0.41–2.26	<0.001	88%	0.92
Hemoptysis	1	17/632 (2.7%)	14/1,283 (1.1%)	2.51	1.23–5.12	NA	NA	**0.01**
RBC transfusion	5	2,328/24,894 (9.6%)	5,587/38,388 (14.6%)	0.54	0.37–0.79	0.05	57%	**0.002**

*CI, confidence interval; ECMO, extracorporeal life support; ICU, intensive care unit; IVA, inferior vena cava; NA, not applicable; OR, odds ratio; RBC, red blood cell.*

*Statistically significant differences are marked bold.*

Ninety-day mortality in the CDT group was 6.1% compared to 9.7% for ST group (OR = 0.66; 95% CI: 0.19–2.28, *I*^2^ = 0%, *p* = 0.51). Sub-analysis depending on the study design showed also no statistically significant differences between CDT and ST groups in RCT (11.1 vs. 14.0%, respectively; OR = 0.77, 95% CI: 0.20–2.98, *p* = 0.71) and in non-RCT (0.0 vs. 3.4%, respectively; OR = 0.31, 95% CI: 0.01–7.96, *p* = 0.48).

One-year mortality was reported in two studies and was also lower in CDT group, compared to ST group (11.9 vs. 19.4%; OR = 0.46, 95% CI 0.24–0.87, *I*^2^ = 61%, *p* = 0.02).

### Secondary Outcomes

Regarding complications, the rates of myocardial injury, cardiac arrest, and stroke were lower in CDT group, compared to ST group (4.3 vs. 5.9% for AMI; 3.9 vs. 11.2% for cardiac arrest; 2.6 vs. 5.9% for stroke; *p* < 0.001 for all). In addition, patients treated with CDT had lower readmission rate at 30 days, compared to those treated with ST (6.9 vs. 9.6%, *p* < 0.001). The rates of acute kidney injury and complete heart block were comparable between the groups.

Bleeding complications occurred more often in CDT group, compared to ST (13.6 vs. 9.5%; *p* = 0.002). However, the rates of any major bleeding, hemoptysis, and red blood cell (RBC) transfusion were lower in patients treated with CDT, compared to ST (11.9 vs. 17.4% for major bleeding, 2.7 vs. 1.1% for hemoptysis, and 9.6 vs. 14.6% for RBC transfusion; *p* ≤ 0.01 for all). The rate of intracranial hemorrhage and gastrointestinal bleeding was comparable in both groups.

**FIGURE 2:**
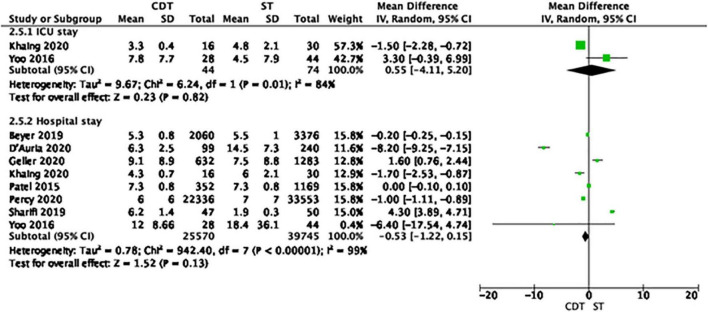
Forest plot of length of hospital stay in the catheter-directed therapies (CDT) group and systemic thrombolysis (ST). Each green rectangle or square represents an individual study, with 95% confidence intervals of the result displayed as black horizontal lines. The diamonds show the result when all the individual studies are combined together and averaged.

Regarding the use of hospital resources, ECLS was used more often in patients treated with CDT, compared to ST (0.5 vs. 0.2%, OR = 2.52, 95% CI 1.88–3.39, *I*^2^ = 0%, *p* < 0.001). The rates of ICU admission, invasive mechanical ventilation, and IVC filter insertion were comparable in both groups. The length of hospital stay was also comparable in patients treated with CDT and ST (Figure 2).

## Discussion

In this meta-analysis of 11 studies including 65,589 patients with intermediate-high/submassive and high-risk/massive PE, we found patients treated with CDT had lower in-hospital, 30-day and 1-year mortality, less in-hospital complications (AMI, cardiac arrest, stroke, and major bleeding), and lower 30-day readmission rate, compared to those treated with ST. The novelty of our study includes (i) the largest hitherto analyzed a population of 65,589 patients treated with CDT or ST, (ii) the longest follow-up, compared to previous meta-analyses (23, 24), and (iii) inclusion of studies mostly published in the last 2 years, not considered in the previous meta-analyses.

According to the European Society of Cardiology (ESC) guidelines, percutaneous CDT should be considered for patients with high-risk PE, in whom thrombolysis is contraindicated or has failed (class of recommendation IIa, level of evidence C) (2). Although ST remains the mainstay of treatment in intermediate-high and high-risk PE patients, it is associated with relatively high risk of major bleeding (10%) and intracranial bleeding (3–5%) (3, 25). To circumvent bleeding complications, catheter-directed delivery of a low-dose thrombolytic agent has been proposed as safer and more efficient when compared to ST, thus giving rise to the dynamic development of CDT as an alternative to ST in the last decade and shifting paradigms regarding PE management (23). In contrary to the ESC guidelines, CDT were recently suggested not only as an alternative, but also as a first-line treatment strategy in hemodynamically unstable patients with acute PE (26). The feasibility of alternative approaches for CDT has also been suggested as attractive for the treatment of high and intermediate-high PE, such as transcubital venous access (27).

The results of trials comparing ST and catheter-directed thrombolysis are controversial, with some trials suggesting better outcomes (7, 13, 14, 16, 17), and others showing comparable (15, 18, 20–22) or worse outcomes after catheter-directed thrombolysis, compared to ST (19, 28, 29). Recent evidence also showed comparable efficacy and safety of catheter-directed thrombolysis with and without ultrasound, both in intermediate and high-risk groups (8, 30, 31). Since mechanical thrombectomy is performed mostly without the use of thrombolytic agents, it has been hypothesized to reduce major bleeding rates compared with both ST and catheter-directed thrombolysis, especially in intermediate-risk patients. However, the clinical efficacy of mechanical thrombectomy has been demonstrated only in single-arm trials through a surrogate endpoint (reduction of the right ventricle/left ventricle ratio at 48-h follow-up), warranting caution when interpreting the results (32, 33). Hence, the best interventional treatment option in patients with PE remains to be established (34). For this reason, we did not focus on a specific CDT type, but included all CDT in our analysis (Table 1). In contrast to previous recent analyses (35, 36), we compared the techniques which require the involvement of the cath-lab team and specialist equipment against systemic thrombolysis, which can be readily administered in the emergency room. Hence, our analysis was dictated by the practical and organizational considerations.

Compared to a similar meta-analysis published in 2009, the in-hospital mortality decreased over twofold, from 13.6 to 6.4% (23). The decrease in early mortality might be due to two reasons. First, the dynamic development of CDT techniques, along with the increasing experience of the operators, might contribute to better long-term outcomes (37). Second, establishment of pulmonary embolism response teams (PERTs) facilitates the early risk stratification and appropriate patient selection for CDT *via* multidisciplinary approaches (38). Although the information regarding PERT activity has only been reported in two out of 11 studies, we could not perform any reliable sub-analysis regarding the effect of PERT on patient outcomes. However, preliminary evidence from other trials suggests that PERT implementation improved the time to treatment initiation and decreased both the hospital length of stay and the generated costs (39–41). Thus, the technological, operational, and organizational improvements likely all contribute to better early outcomes in patients with intermediate and high-risk PE.

The mortality at 1 year had not previously been considered in a meta-analysis. However, the 1-year mortality observed in our study (11.9% in the CDT group and 19.4% in the ST group) is comparable with 1-year mortality rates reported in population-based cohort studies (16.8–16.9%) (42, 43). Since data regarding 1-year mortality have only reported in two out of 11 studies included in our analysis, these results should be interpreted with caution and re-evaluated in future trials.

We found a lower rate of in-hospital complications (AMI, cardiac arrest, and stroke) in patients treated with CDT, compared with ST. However, considering that these outcomes were reported only in one study, they should be perceived as hypothesis generating. In our study, CDT was associated with a higher rate of any bleeding (13.6 vs. 9.5%), confirming previous findings (19, 28, 29). Likely, the bleeding events related to CDT are associated with the use of anticoagulants prior to CDT. However, in the absence of individual patient data regarding previous anticoagulation, we could not perform a subgroup analysis to determine the association between anticoagulation before CDT and bleeding risk afterward. The rate of major bleeding and the need for RBC transfusion were lower in CDT than in ST (11.9 vs. 17.4% and 9.6 vs. 14.6%, respectively) and comparable to the major bleeding rate reported in observational studies (13–22.8%) (41, 43), confirming higher safety of CDT relative to ST. Noteworthy, in a network meta-analysis comparing catheter-directed thrombolysis, mechanical thrombectomy, and ST, mechanical thrombectomy was associated with the lowest rate of major bleeding, compared to any other interventional treatment method (24). Further dedicated trials are needed to determine the bleeding risk associated with different CDT.

In our meta-analysis, CDT were associated with comparable use of hospital resources, including ICU admission, invasive mechanical ventilation and IVC filter insertion, comparable length of hospital state, and higher rate of ECLS application. On the contrary, CDT were associated with lower readmission rate, which could theoretically decrease the healthcare costs related to the acute PE episode. However, in the absence of the dedicated economic analysis, we cannot draw any firm conclusion regarding the cost-effectiveness of CDT, compared to ST. Evidence from previous studies showed that CDT might be a more cost-effective treatment strategy compared to ST, despite higher costs of initial hospitalization (16, 21).

Altogether, our analysis suggests that CDT may have the potential to reduce mortality and complications following intermediate and high-risk PE. Our results should be interpreted with caution due to significant heterogeneity between the trials, which is due to differences in study populations (intermediate-high and high-risk PE), CDT (with or without thrombolysis), outcome measured, and duration of follow-up. Future high-quality evidence from randomized controlled trials is required to provide clarity regarding the optimal PE management in specific subgroups of patients, including (i) the efficacy and safety of various CDT therapies among each other and relative to ST, (ii) the best therapy dependent on the patient’s risk of mortality (intermediate vs. high), and (iii) the effect of PERT implementation on clinical outcomes. For example, the Pulmonary Embolism International THrOmbolysis (PEITHO)-3 study (NCT04430569) will investigate whether reduced-dose thrombolysis improves safety while maintaining reperfusion efficacy compared with standard heparin anticoagulation in 650 patients with intermediate-high-risk PE in a randomized, placebo-controlled, double-blind manner (44).

## Limitations

Our analysis has several limitations. First and foremost, it combines two essentially different groups of patients with high-risk (massive) PE and intermediate-high-risk (submassive) PE. The risk of death in both groups is substantially different (40 vs. 5–10%). Accordingly, the main treatment goal in patients with high-risk PE is to achieve the prompt reduction in pulmonary artery pressure and resistance, with a concomitant improvement in RV function, whereas the main goal in patients with intermediate-high-risk PE is to prevent the hemodynamic breakdown (2). The combination of different groups of patients and study designs resulted in significant heterogeneity between the trials, requiring very cautious interpretation of the results. Ideally, both groups of patients should be analyzed separately to derive firm conclusion regarding the efficacy and safety of CDT depending on patients’ clinical presentation. However, since the percentage of patients with submassive and massive PE has only been reported in two out of 11 articles included in this analysis (13, 22), such sub-analysis was not feasible. Similarly, a sub-analysis of patients hemodynamically stable or unstable at admission, or those treated with different CDT, could not be done either, since such data were not available in the individual manuscripts. Second, our analysis is mostly based on retrospective studies that differed regarding geographical setting and baseline characteristics, which might have affected the results. Third, we were unable to access individual patient data, such as time from hospital admission to administration of ST or CDT, fibrinolytic agent (alteplase, reteplase, or tenecteplase) and dose (full or half), the use of anticoagulation prior to CDT, detailed PE risk stratification, or co-existent neoplastic disease. Hence, the potential influence of these confounders might potentially have affected the outcomes. Fourth, based on the design of the studies included in this meta-analysis, we could not perform separate comparative analyses of different CDT, including catheter-directed thrombolysis, ultrasound-assisted thrombolysis, and mechanical thrombectomy. Fifth, some of the outcomes such as AMI, cardiac arrest, or stroke have only been reported in one or two studies, which limits the general applicability of the results. Nonetheless, our systematic review with meta-analysis has been performed according to the Cochrane’s methodology and presents data derived from the hitherto largest population of PE patients treated with CDT or ST, with the longest follow-up, compared to previous meta-analyses. Finally, the protocol of this meta-analysis was not pre-registered in PROSPERO due to a significant delay caused by the priority of the research related to COVID-19, according to the information obtained from PROSPERO.

## Conclusion

Our meta-analysis showed that CDT might decrease mortality in patients with intermediate-high and high-risk pulmonary embolism and were associated with less complications, including major bleeding, and lower 30-day readmission rate. Our results support further randomized trials to investigate the safety and efficacy use of CDT in patients with PE.

## Data Availability Statement

The original contributions presented in the study are included in the article/Supplementary Material, further inquiries can be directed to the corresponding author.

## Author Contributions

AP and AG: conceptualization, methodology, and supervision. ŁS, AG, and AP: validation, investigation, and data curation. MP and TK: formal analysis. MG and MK: resources. AP: writing—original draft preparation and project administration. AP, AG, ŁS, and MN: writing—review and editing. SD, AG, and MB: visualization. All authors have been read and approved the manuscript for submission to JTH.

## Conflict of Interest

The authors declare that the research was conducted in the absence of any commercial or financial relationships that could be construed as a potential conflict of interest.

## Publisher’s Note

All claims expressed in this article are solely those of the authors and do not necessarily represent those of their affiliated organizations, or those of the publisher, the editors and the reviewers. Any product that may be evaluated in this article, or claim that may be made by its manufacturer, is not guaranteed or endorsed by the publisher.
