# Supplementary material for: Catheter-Based Therapies Decrease Mortality in Patients With Intermediate and High-Risk Pulmonary Embolism: Evidence From Meta-Analysis of 65,589 Patients
Source: Front Cardiovasc Med. 2022 Jun 16;9:861307. doi: 10.3389/fcvm.2022.861307 (PMC9243366; doi:10.3389/fcvm.2022.861307)
Supplement: Supplementary file 1 [file Data_Sheet_1.DOCX]

**Supplementary material**

**Table S1**. Study inclusion and exclusion criteria, primary outcomes and findings.

| **Study** | **Inclusion criteria** | **Exclusion criteria** | **Primary outcome(s)** | **Finding(s)** |
| --- | --- | --- | --- | --- |
| Beyer et al. 2019 | Patients aged >18 years hospitalized due to acute, non-septic PE according to ICD-10-CM and treated with any form of thrombolysis according to ICD-10-PCS. | Hospitalization in a state other than the patient’s primary residence (any readmission occurring in a different state from that the index hospitalization was not be indexed in the database). | In-hospital mortality and 30-day readmission rate. | ST was used more often than CDT in patients with acute PE, in particular among those with high-risk features. Among patients treated with CDT, there were no differences in events between CDT-US and CDT alone. |
| D’Auria et al. 2020 | A submassive PE on presentation leading to admission to the ICU within the first day of admission. | Patients discharged from an acute care facility within five days preceding the PE admission; those who underwent surgical or mechanical thrombectomy; those who were discharged from the index admission into a hospice. | All-cause mortality. | In patients presenting with acute submassive pulmonary embolism who are admitted to an intensive care unit, the group treated with CDT had reduced mortality at 30 days and 1 year when compared to medical therapy, without increase in bleeding. |
| Geller et al. 2020 | Patients hospitalized with PE | NS | Intracranial hemorrhage (ICH) | CDT was associated with similar ICH rates, increased all-cause bleeding, and lower short and intermediate-term mortality compared with ST. |
| Harrison et al. 2021 | Patients aged >65 years with a diagnosis of PE from the Pulmonary Embolism Response Team database. | Patients who previously underwent systemic thrombolysis, mechanical and surgical embolectomy | NS | Elderly patients have similar in-hospital mortality and length of stary when treated with CDT versus anticoagulation alone. In elderly patients who have high-to- intermediate risk acute PE and are candidates for interventional therapy, CDT may be a safe alternative treatment. |
| Hennemeyer et al. 2018 | Acute massive or submassive  pulmonary embolism diagnosed  with computed tomography angiography (CTA); evidence of right heart strain on CTA, echocardiogram, or magnetic resonance imaging; completion of catheter-based therapy if initiated; follow-up imaging 24-48 hours after treatment. | Failure to complete catheter treatment if initiated, the presence of septic emboli, or patients previously treated with systemic thrombolysis. | Improvement of right ventricle/ left ventricle ratio (RV/LV) 24-48 hours after treatment | CDT for acute massive and submassive pulmonary embolism significantly improves RV/ LV ratio at 24-48 hours compared with anticoagulation alone and may lower hospital readmission rates. CDT may be more advantageous in patients with mild to moderate RV dilation. |
| Khaing et al. 2020 | Case of PE based on a transthoracic echocardiogram (TTE) showing a clot-in-transit. | NS | Need for admission to the intensive care unit (ICU) at any point during the hospitalization | Adoption of the PERT model at an academic tertiary care center was associated with low rates of mortality and bleeding, similar to other published studies. CDT in select patients under PERT consultation may be associated with shorter ICU and hospital length of stay. |
| Kucher et al. 2014 | Acute symptomatic PE confirmed by contrast-enhanced CTA with embolus located in at least 1 main or proximal lower lobe pulmonary artery RV/LV ratio ≥1 obtained from TTE apical 4-chamber view. | Age <18 or >80 years; index PE symptom duration >14 days; insufficient TTE quality; known significant bleeding risk; administration of thrombolytic agents within the previous 4 days; active bleeding; known bleeding diathesis; known coagulation disorder; platelet count <100 000/mm3; previous use of vitamin K antagonists with international normalized ratio >2.5 on admission; history of intracranial or intraspinal surgery or trauma or bleeding; intracranial neoplasm, arteriovenous malformation or aneurysm; gastrointestinal bleeding <3 months; internal eye surgery or hemorrhagic retinopathy <3 months; major surgery, cataract surgery, trauma, obstetric delivery, cardiopulmonary resuscitation, or other invasive procedure <10 days; allergy, hypersensitivity, or thrombocytopenia from heparin or alteplase; severe contrast allergy to iodinated contrast; known right-to-left cardiac shunt (e.g., from a large patent foramen ovale or atrial septal defect); large (>10 mm) right atrial or RV thrombus; hemodynamic decompensation; severe hypertension on repeated readings (systolic >180 mm Hg or diastolic >105 mmHg); pregnancy, lactation, or parturition <30 days; participation in any other investigational drug or device study; life expectancy <90 days; and inability to comply with study assessments. | The difference in the RV/ LV ratio from baseline to 24 hours, evaluated by the blinded core laboratory. | In patients with pulmonary embolism at intermediate risk, a standardized USAT regimen was superior to anticoagulation with heparin alone in reversing RV dilatation at 24 hours, without an increase in bleeding complications. |
| Patel et al. 2015 | Adult patients with 9th Revision Clinical Modification (ICD-9-CM) diagnostic code 415.11(Iatrogenic PE and infarction), 415.13 (Saddle embolus of pulmonary artery), and 415.19 (Other PE and infarction) | Patients aged < 18 years. | In-hospital mortality | CDT was associated with lower in-hospital mortality and combined in-hospital mortality and ICH. |
| Percy et al. 2020 | All patients undergoing SE, ST, or CDT for a primary diagnosis of acute PE. | Patients aged < 18 years. | All-cause in-hospital mortality. | Mortality occurred in 19.8% of patients undergoing surgical embolectomy for acute pulmonary embolism. This represents a significant improvement compared with traditional outcomes and supports the role of surgery in the multidisciplinary treatment of this high-risk condition. |
| Sharifi et al. 2019 | Patients who underwent UFCDT or HDT for massive and submassive PE | NS | Death, major bleeding, recurrence of venous thromboembolism (VTE), and trans- fusion of packed red blood cells as a result of bleeding. | Both CDT and half-dose ST lead to rapid reduction of pulmonary artery systolic pressure and RV/LV, whereas half-dose ST leads to a lower duration and cost of hospitalization. |
| Yoo et al. 2016 | Patients aged > 18 years diagnosed with acute massive or submassive PE and treated with either ST or CDT as a rescue therapy. | NS | Mortality at 7, 14, or 28 days. | Similar clinical outcomes were shown between ST and CDT in patients with acute massive or submassive PE. |

**Table S2.** Past medical history of patients treated with catheter-directed therapies (CDT) and systemic thrombolysis (ST)

| **Comorbidity type** | **No of studies** | **Events/participants** | | **Outcome differences** | | **Heterogeneity between trials** | | **P-value for**  **differences**  **across groups** |
| --- | --- | --- | --- | --- | --- | --- | --- | --- |
|  |  | **CDT group** | **ST group** | **OR^[[1]](#endnote-1)^** | **95%CI^[[2]](#endnote-2)^** | **P-value** | **I^2^ statistic** |  |
| Hypertension | 8 | 13,025/25,521 (51.0%) | 19,182/39,547 (48.5%) | 1.02 | 0.89 – 1.17 | 0.03 | 54% | 0.78 |
| Diabetes mellitus | 8 | 5,141/23,479 (21.9%) | 8,341/36,279 (23.0%) | 0.95 | 0.92 – 0.99 | 0.64 | 0% | **0.02** |
| Shock | 2 | 192/2,412 (8.0%) | 782/4,545 (17.2%) | 0.48 | 0.32 – 0.73 | 0.03 | 79% | **<0.001** |
| DVT^[[3]](#endnote-3)^ | 6 | 1,325/2,795 (47.4%) | 2,190/4,873 (44.9%) | 1.15 | 0.83 – 1.59 | 0.03 | 59% | 0.40 |
| Cardiac arrest | 3 | 104/2,440 (4.3%) | 609/4,589 (13.3%) | 0.45 | 0.21 – 0.97 | <0.001 | 86% | **0.04** |
| Congestive heart failure | 4 | 3,101/23,097 (13.4%) | 4,795/35,105 (13.7%) | 0.98 | 0.94 – 1.03 | 0.47 | 0% | 0.53 |
| Coronary artery disease | 4 | 3,594/22,501 (16.0%) | 4,197/33,865 (12.4%) | 1.01 | 0.59 – 1.73 | 0.14 | 44% | 0.96 |
| Chronic pulmonary disease | 6 | 4,986/23,453 (21.3%) | 6,903/36,383 (19.0%) | 0.91 | 0.70 – 1.17 | 0.003 | 72% | 0.45 |
| Cancer | 8 | 2,859/22,610 (12.6%) | 3,505/34,097 (10.3%) | 0.50 | 0.23 – 1.10 | <0.001 | 81% | 0.09 |
| Renal failure | 3 | 59/666 (8.9%) | 141/1,421 (9.9%) | 0.92 | 0.67 – 1.27 | 0.75 | 0% | 0.61 |
| Liver disease | 2 | 36/648 (5.6%) | 67/1,313 (5.1%) | 1.10 | 0.72 – 1.66 | NA^[[4]](#endnote-4)^ | NA | 0.67 |
| Previous myocardial infarction | 2 | 3,728/22,968 (16.2%) | 5,547/34,836 (15.9%) | 1.01 | 0.97 – 1.06 | 0.49 | 0% | 0.54 |
| Previous stroke | 4 | 4,419/23,034 (19.2%) | 5,221/34,908 (15.0%) | 1.34 | 1.28 – 1.40 | 0.41 | 0% | **<0.001** |
| Previous PE^[[5]](#endnote-5)^ | 6 | 180/779 (23.1%) | 293/1,543 (19.0%) | 1.22 | 0.77 – 1.92 | 0.22 | 28% | 0.40 |
| Surgery in the past 30 days | 2 | 107/660 (16.2%) | 188/1,327 (14.2%) | 1.17 | 0.90 – 1.51 | 0.77 | 0% | 0.23 |

[insert supplementary figure S1.] Forest plot of age in the catheter-directed therapies (CDT) group and systemic thrombolysis (ST). The center of each square represents the mean difference (MD) for individual trials, and the corresponding horizontal line stands for the 95% confidence interval (CI). The diamonds represent pooled results.

[insert supplementary figure S2.] Forest plot of male gender in the catheter-directed therapies (CDT) group and systemic thrombolysis (ST). The center of each square represents the odds ratio (OR) for individual trials, and the corresponding horizontal line stands for the 95% confidence interval (CI). The diamonds represent pooled results.

[insert supplementary figure S3.] Forest plot of massive pulmonary embolism in the catheter-directed therapies (CDT) group and systemic thrombolysis (ST). The center of each square represents the odds ratio (OR) for individual trials, and the corresponding horizontal line stands for the 95% confidence interval (CI). The diamonds represent pooled results.

[insert supplementary figure S4.] A summary table of authors' judgements for each risk of bias item in non-randomized trials.

[insert supplementary figure S5.] A plot of the distribution of authors' judgements for each risk of bias item in non-randomized trials.

[insert supplementary figure S6.] A summary table of authors' judgements for each risk of bias item in a randomized trial.

[insert supplementary figure S7.] A plot of the distribution of authors' judgements for each risk of bias item in a randomized trial.

1. odds ratio [↑](#endnote-ref-1)
2. confidence interval [↑](#endnote-ref-2)
3. deep venous thrombosis [↑](#endnote-ref-3)
4. not applicable [↑](#endnote-ref-4)
5. pulmonary embolism [↑](#endnote-ref-5)
