# Supplementary figures and images for: Catheter-Based Therapies Decrease Mortality in Patients With Intermediate and High-Risk Pulmonary Embolism: Evidence From Meta-Analysis of 65,589 Patients
Source: Front Cardiovasc Med. 2022 Jun 16;9:861307. doi: 10.3389/fcvm.2022.861307 (PMC9243366; doi:10.3389/fcvm.2022.861307)

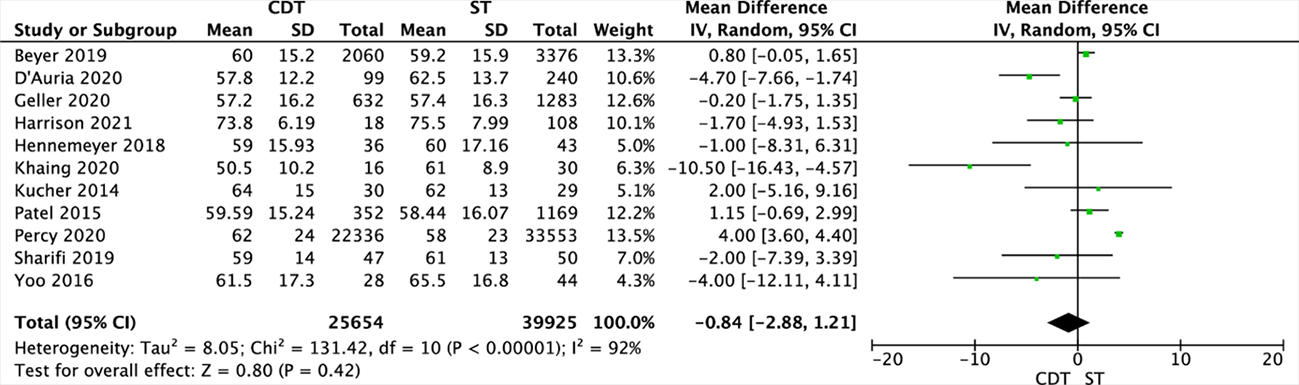

Supplement: Supplementary file 2 [file Image_1.TIF]

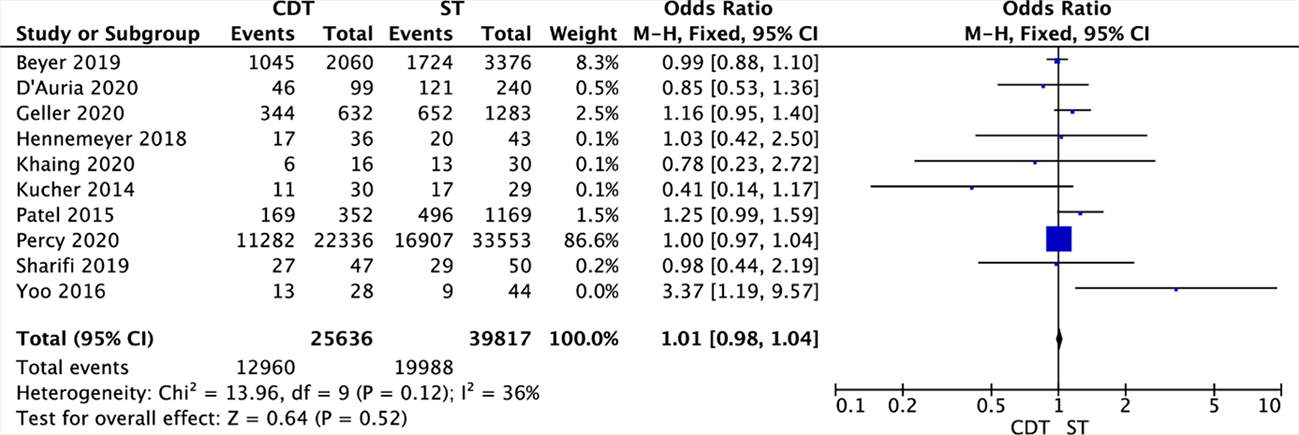

Supplement: Supplementary file 3 [file Image_2.TIF]

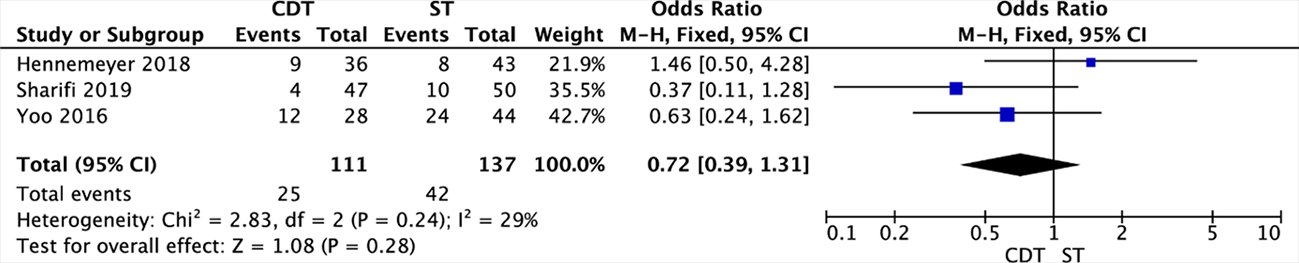

Supplement: Supplementary file 4 [file Image_3.TIF]

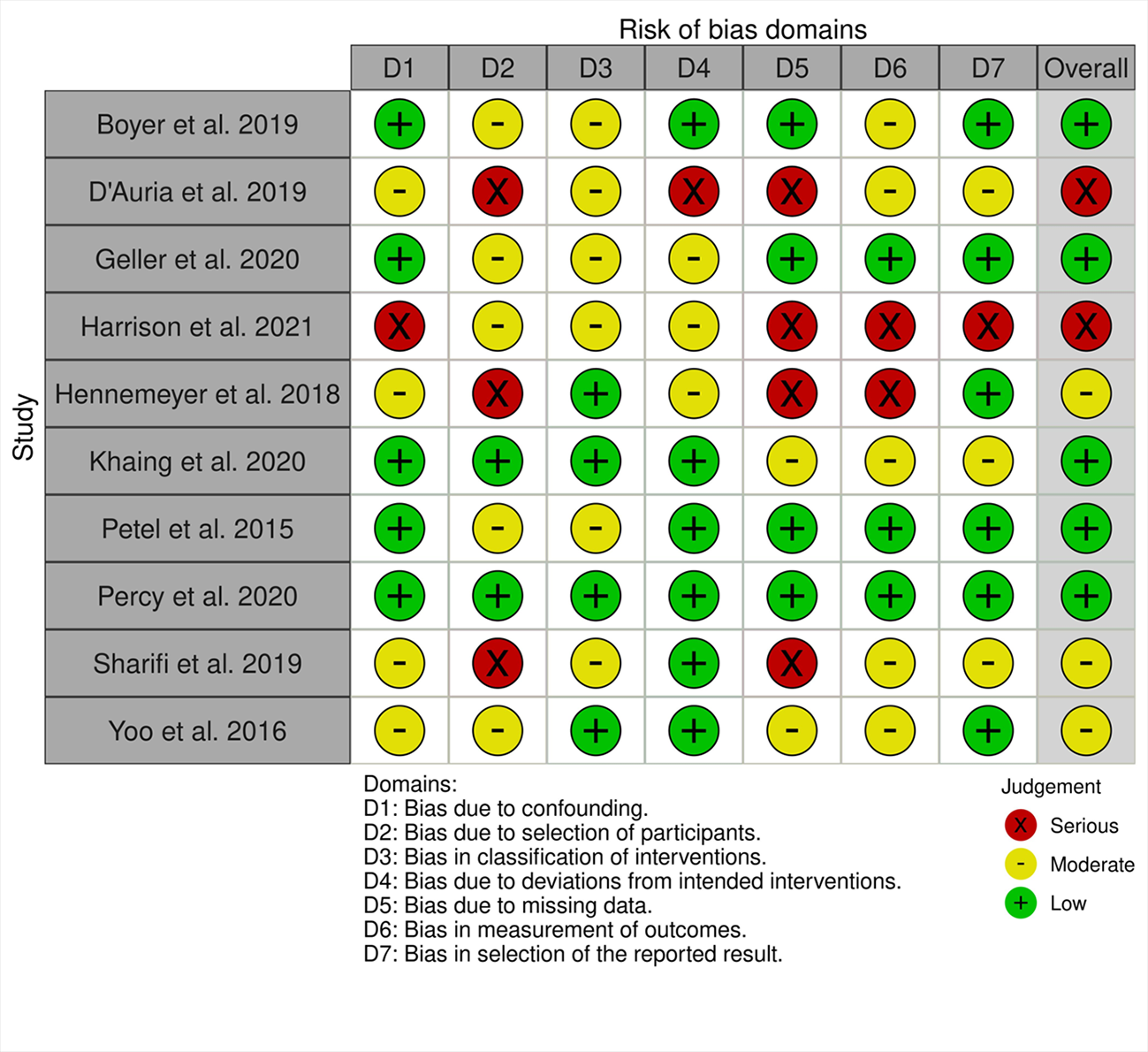

Supplement: Supplementary file 5 [file Image_4.TIF]

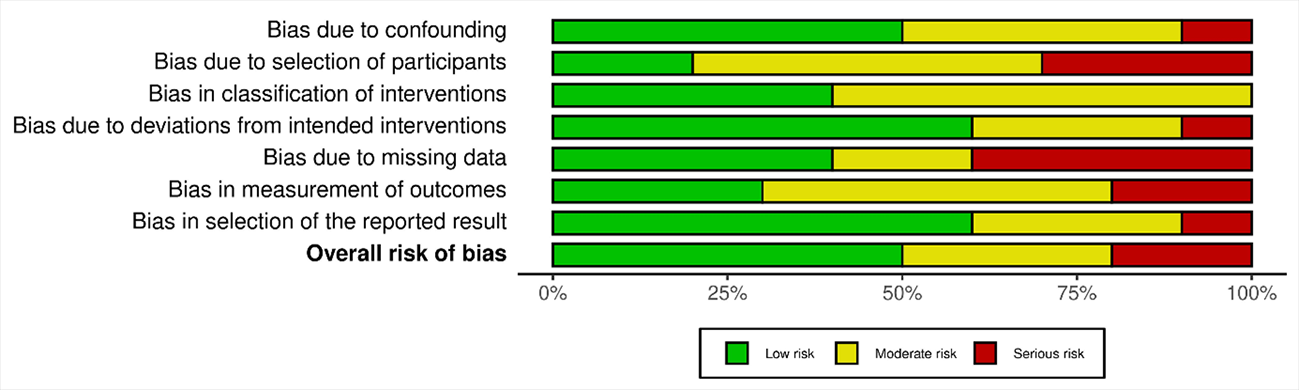

Supplement: Supplementary file 6 [file Image_5.TIF]

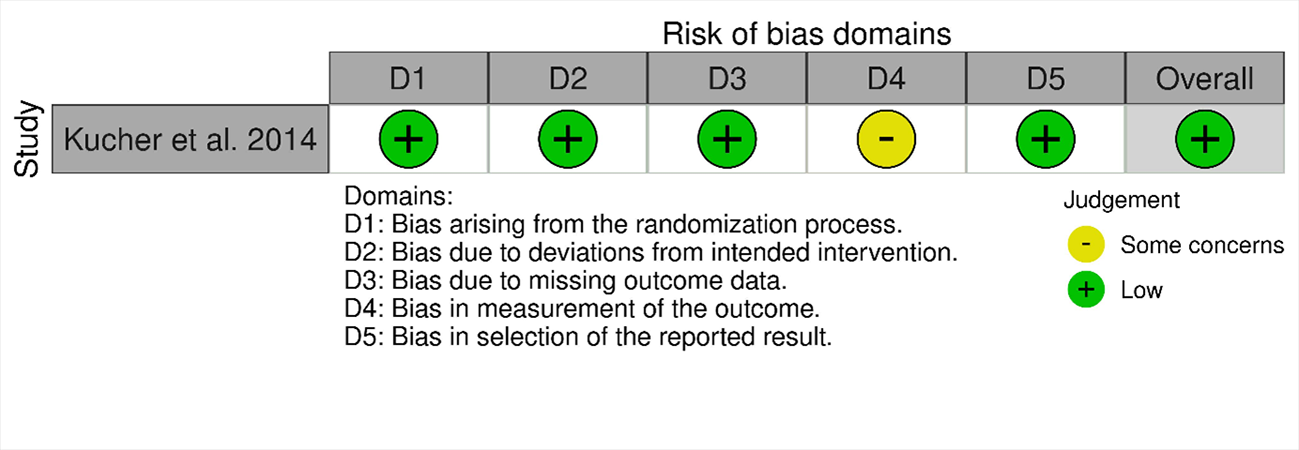

Supplement: Supplementary file 7 [file Image_6.TIF]

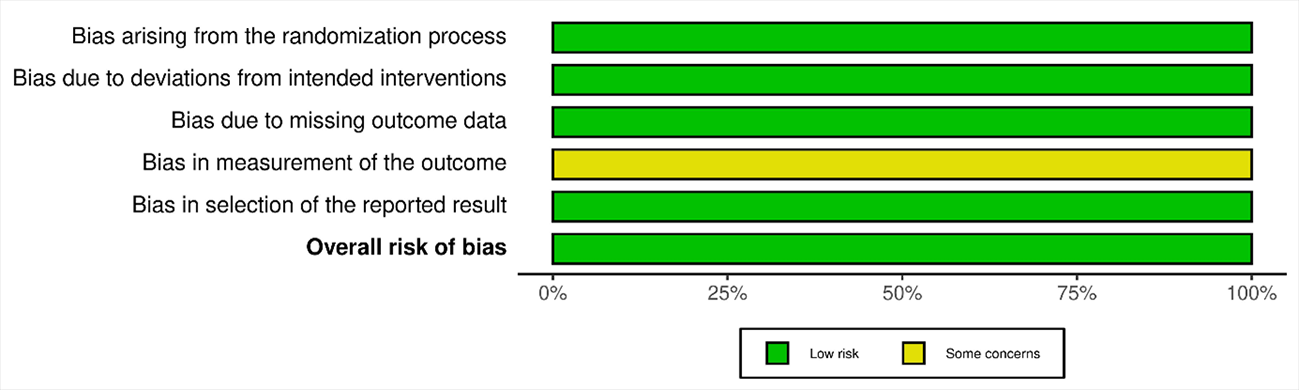

Supplement: Supplementary file 8 [file Image_7.TIF]
